# Supplementary material for: Design, Synthesis and Antibacterial Activity of Coumarin-1,2,3-triazole Hybrids Obtained from Natural Furocoumarin Peucedanin
Source: Molecules. 2019 Jun 5;24(11):2126. doi: 10.3390/molecules24112126 (PMC6600338; doi:10.3390/molecules24112126)

Supplemental Material: ^{1}H and ^{13}C NMR spectra of new compounds.

Design, synthesis and antibacterial activity of coumarin-1,2,3-triazole hybrids obtained from natural furocoumarin peucedanin

Alla V. Lipeeva[1], **Danila O. Zakharov**[1], **Liubov G. Burova**[2], **Tatyana S. Frolova**[3,4], **Dmitry S. Baev**[1], **Ilia V. Shirokikh**[2], **Alexander N. Evstropov**[2], **Olga I. Sinitsyna**[3,4], **Tatyana G. Tolsikova**[1], **Elvira E. Shults**[*1,4]

Affiliations:
[1]Laboratory of Medicinal Chemistry, Novosibirsk Institute of Organic Chemistry, Siberian Branch of the Russian Academy of Sciences, Lavrentyev Ave, 9, 630090 Novosibirsk, Russian Federation;
[2]Department of Microbiology, immunology and virology, Novosibirsk State Medical University, Krasny Prospect 52, 630091, Novosibirsk, Russian Federation;
[3]The Federal Research Center Institute of Cytology and Genetics, Acad. Lavrentyev Ave., 10, 630090, Novosibirsk, Russia;
[4]Novosibirsk State University, Pirogova Str. 2, 630090 Novosibirsk, Russian Federation.

☒ Elvira E. Shults
schultz@nioch.nsc.ru

[1] Laboratory of Medicinal Chemistry, Novosibirsk Institute of Organic Chemistry, Siberian Branch of the Russian Academy of Sciences, Lavrentyev Ave, 9, 630090 Novosibirsk, Russia;

[2] Novosibirsk State University, Pirogova Str. 1, 630090 Novosibirsk, Russia

^{1}H-NMR spectra for compound 4a in $CDCl_3$+CD_3OD

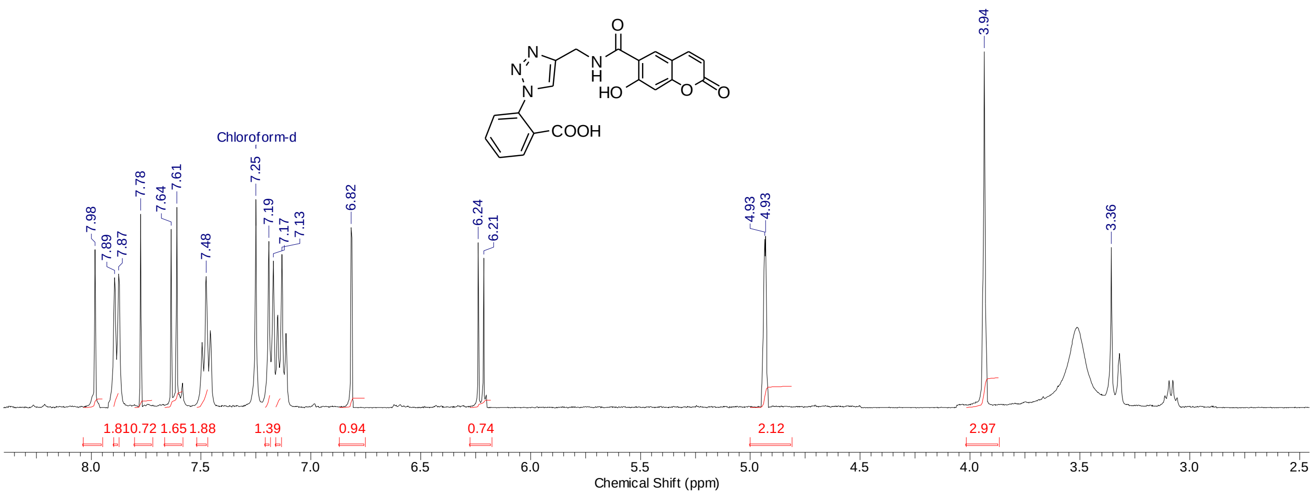

^{13}C-NMR spectra for compound 4a in $CDCl_3$+CD_3OD

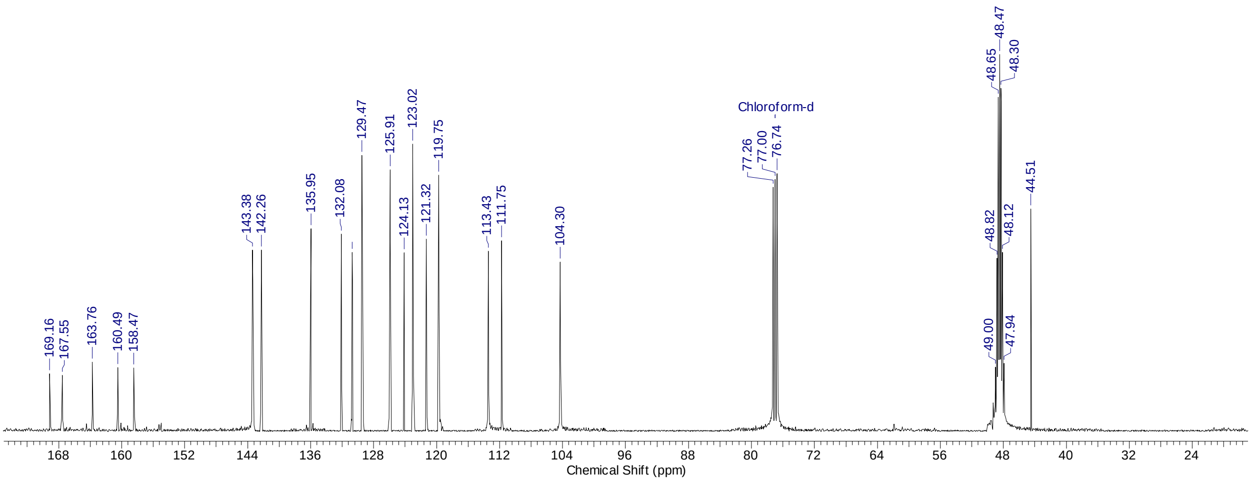

^{1}H-NMR spectra for compound 4b in $CDCl_3$

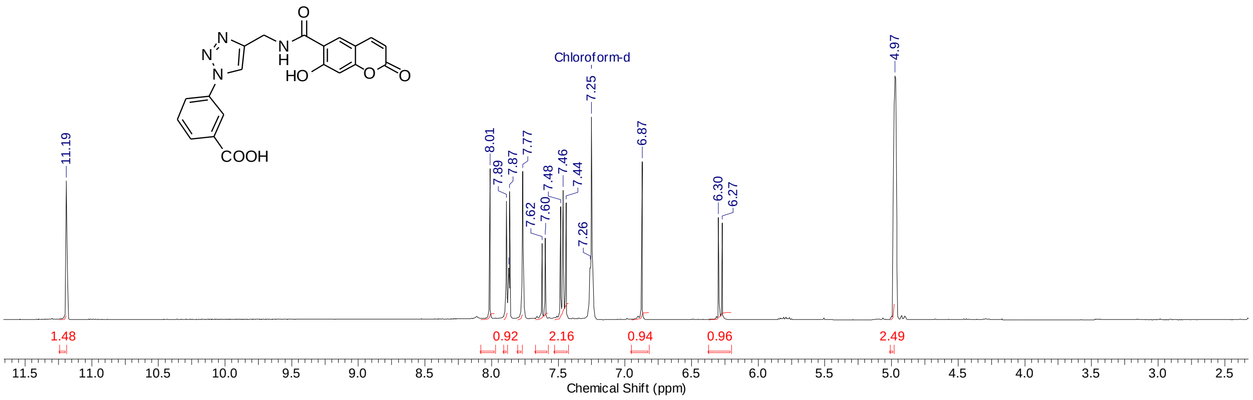

^{13}C-NMR spectra for compound 4b in $CDCl_3$

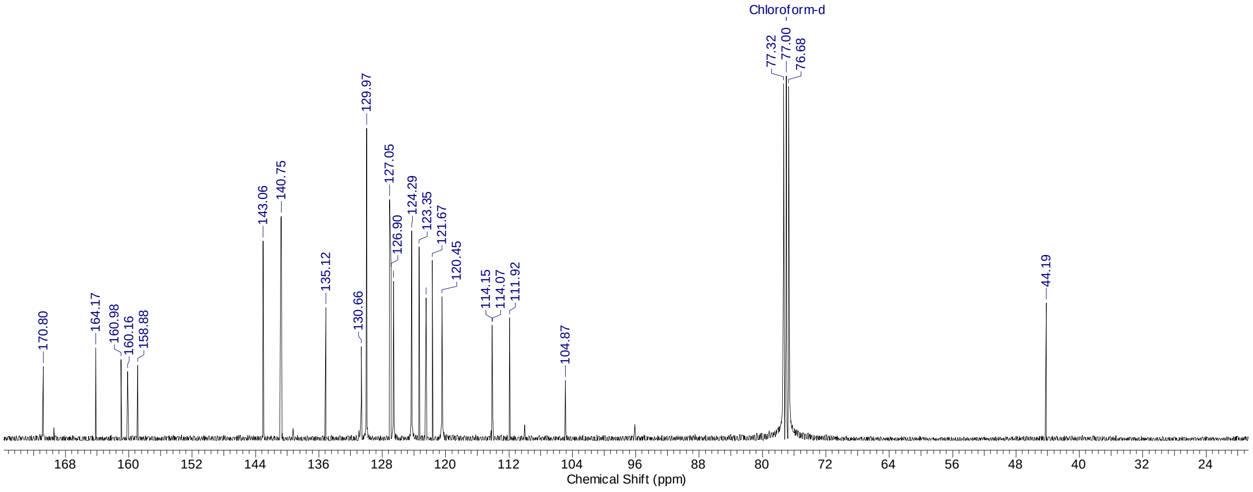

^{1}H-NMR spectra for compound 4c in $CDCl_3$

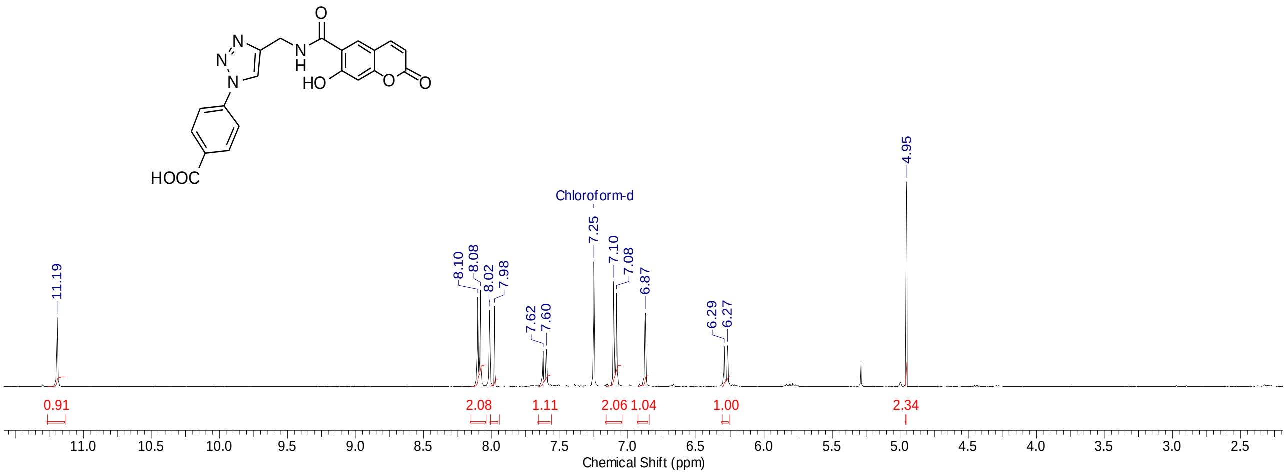

^{13}C-NMR spectra for compound 4c in $CDCl_3$

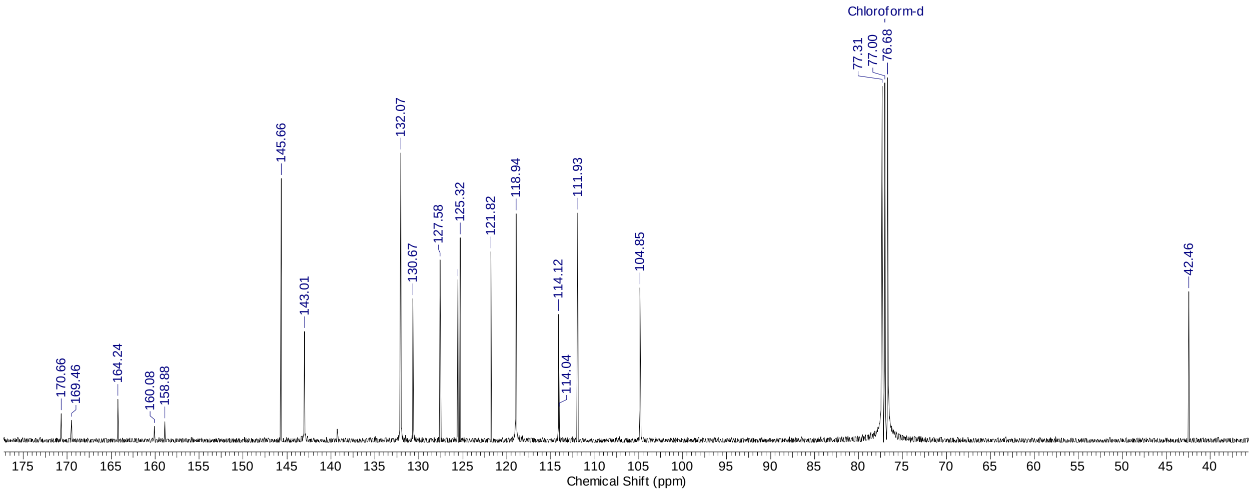

^{1}H-NMR spectra for compound 8 in $CDCl_3$

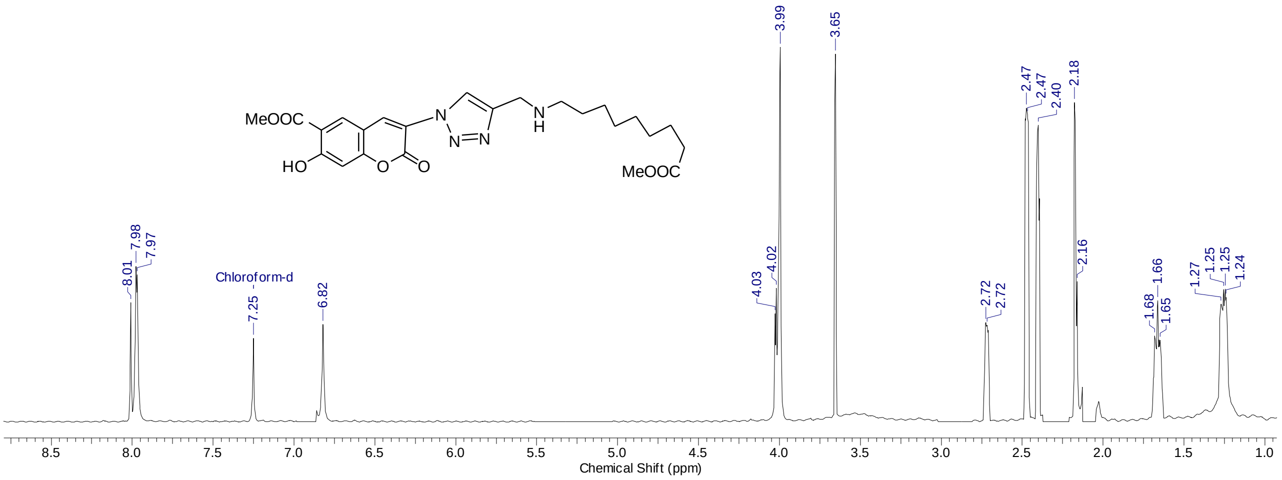

^{13}C-NMR spectra for compound 8 in $CDCl_3$

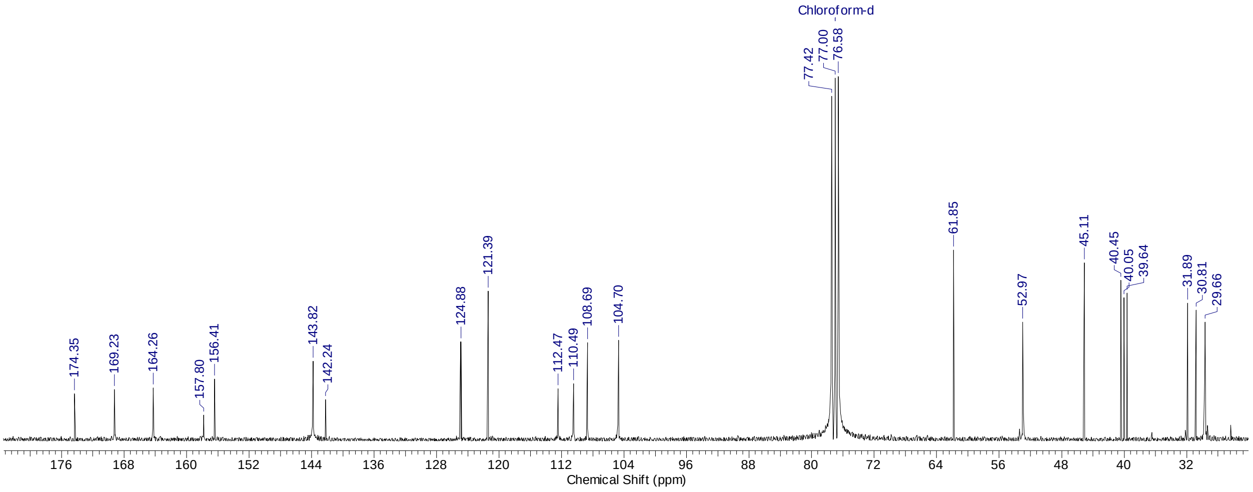

^{1}H-NMR spectra for compound 9 in $CDCl_3$+CD_3OD

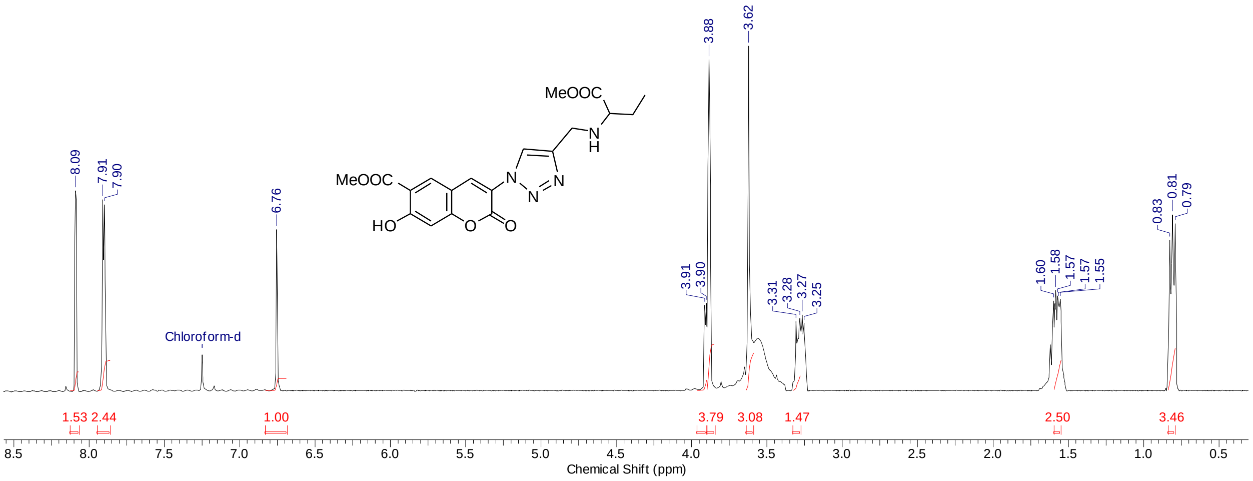

^{13}C-NMR spectra for compound 9 in $CDCl_3$+CD_3OD

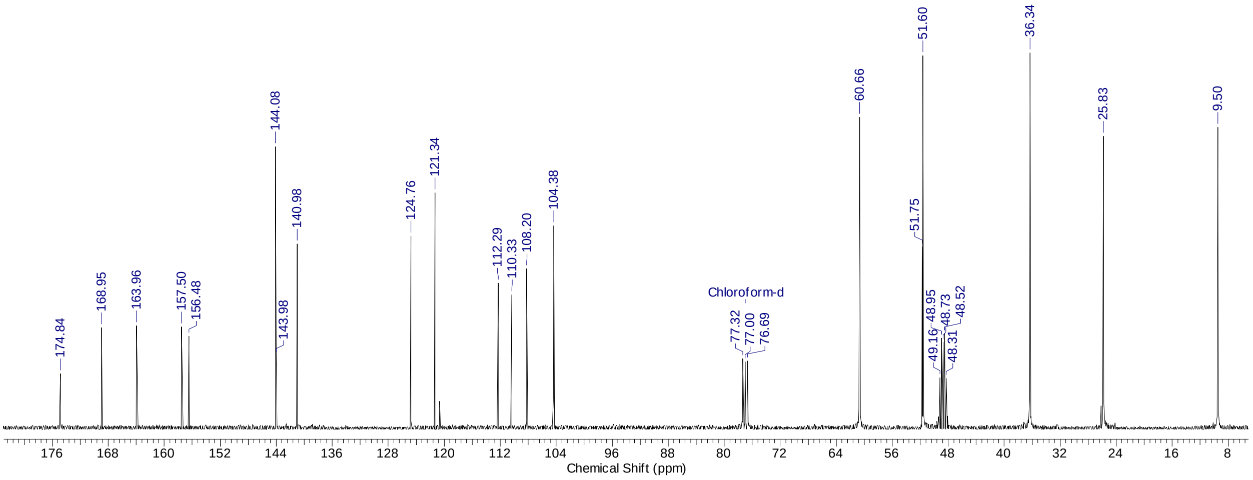

^{1}H-NMR spectra for compound 10 in $CDCl_3$

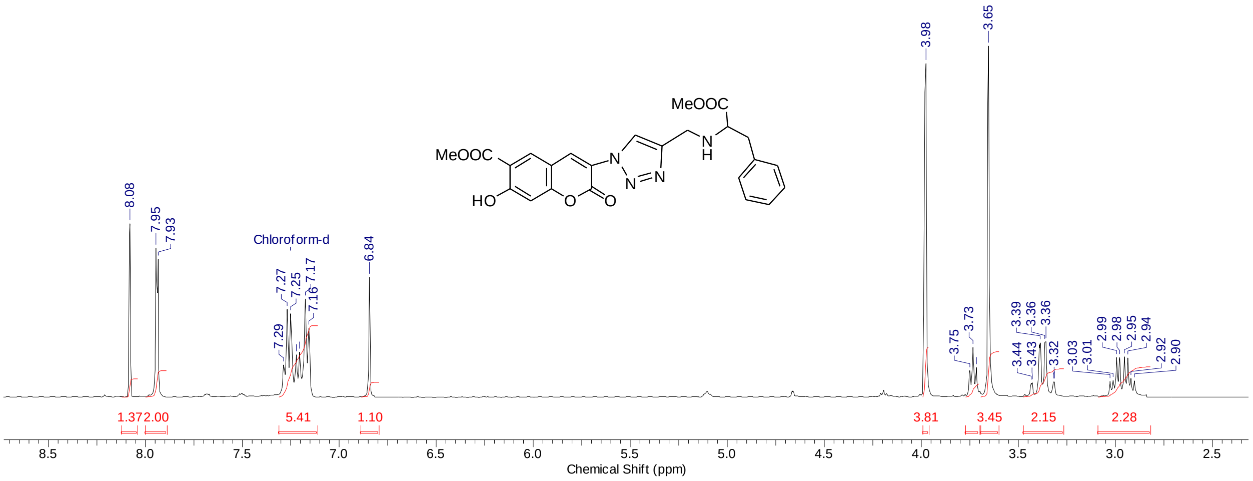

^{13}C-NMR spectra for compound 10 in $CDCl_3$

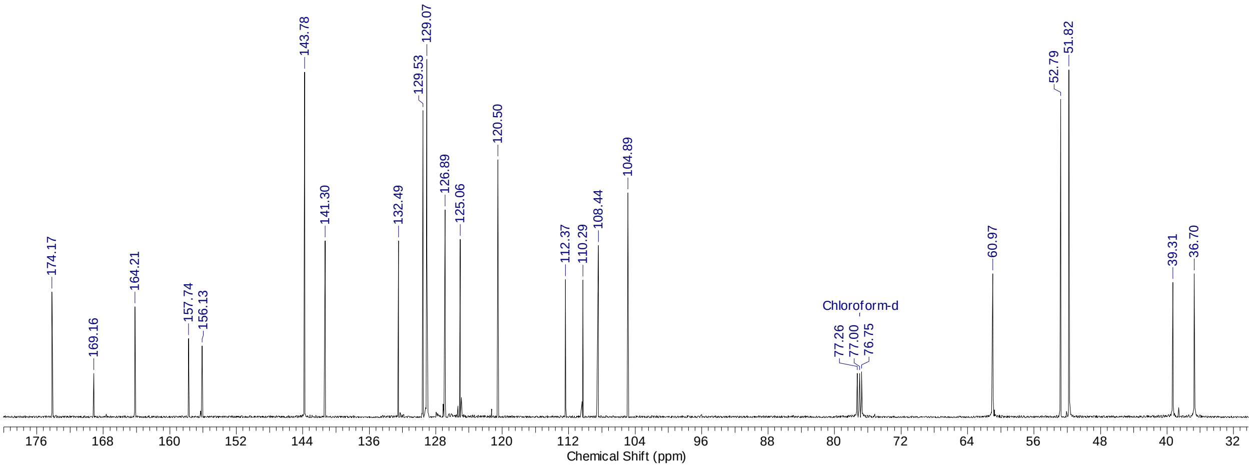

^{1}H-NMR spectra for compound 18 in $CDCl_3$

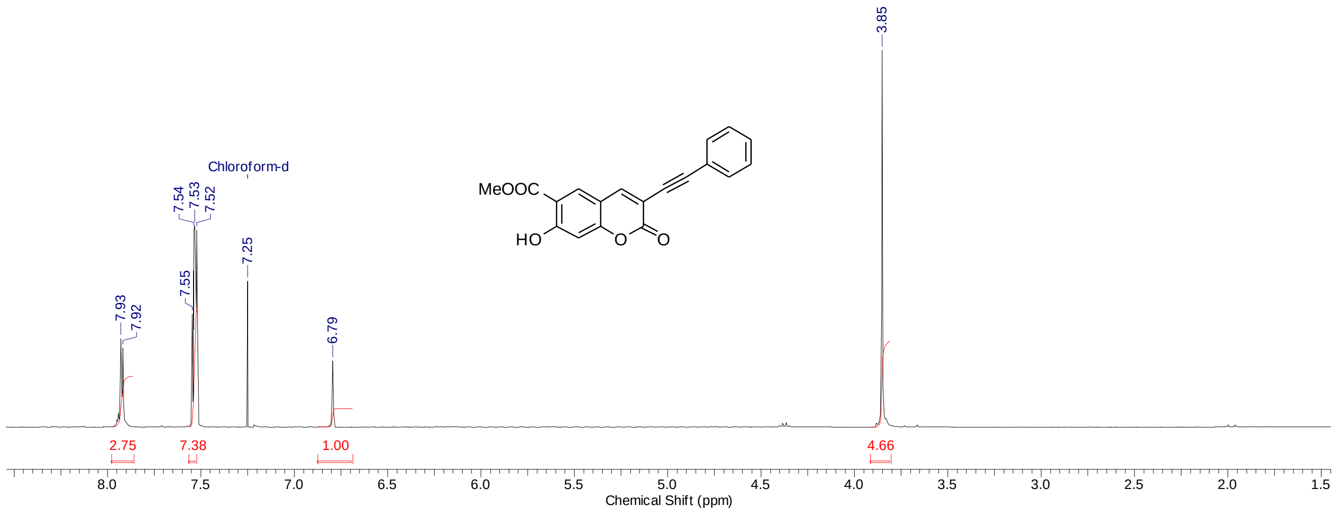

^{13}C-NMR spectra for compound 18 in $CDCl_3$

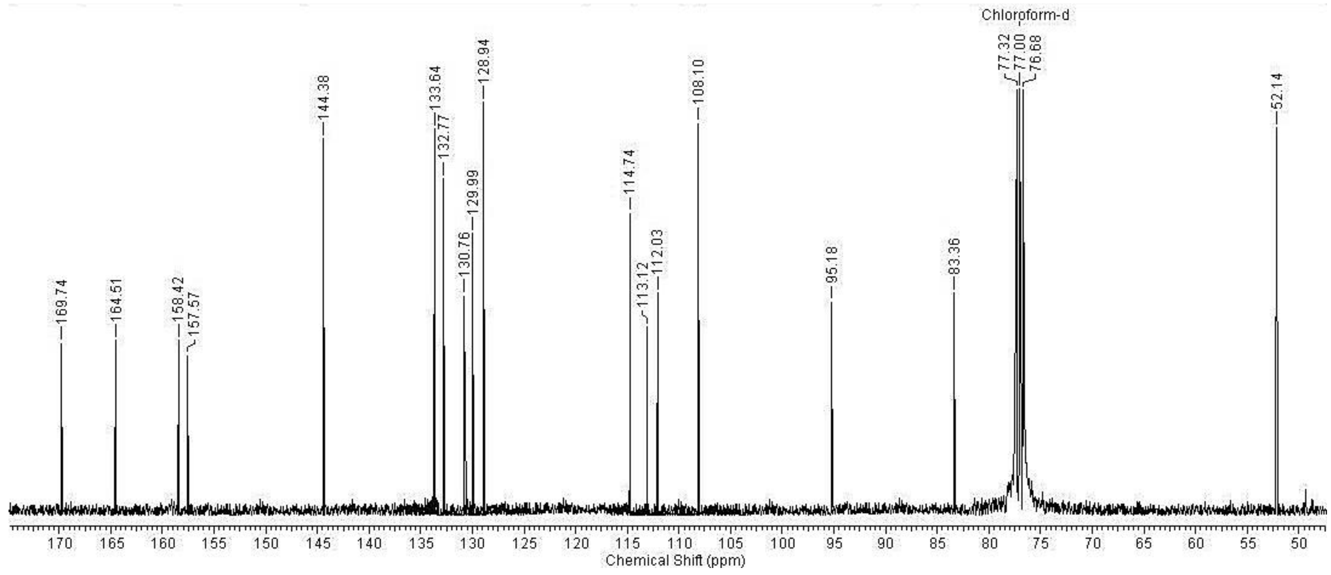

^{1}H-NMR spectra for compound 29 in $CDCl_3$

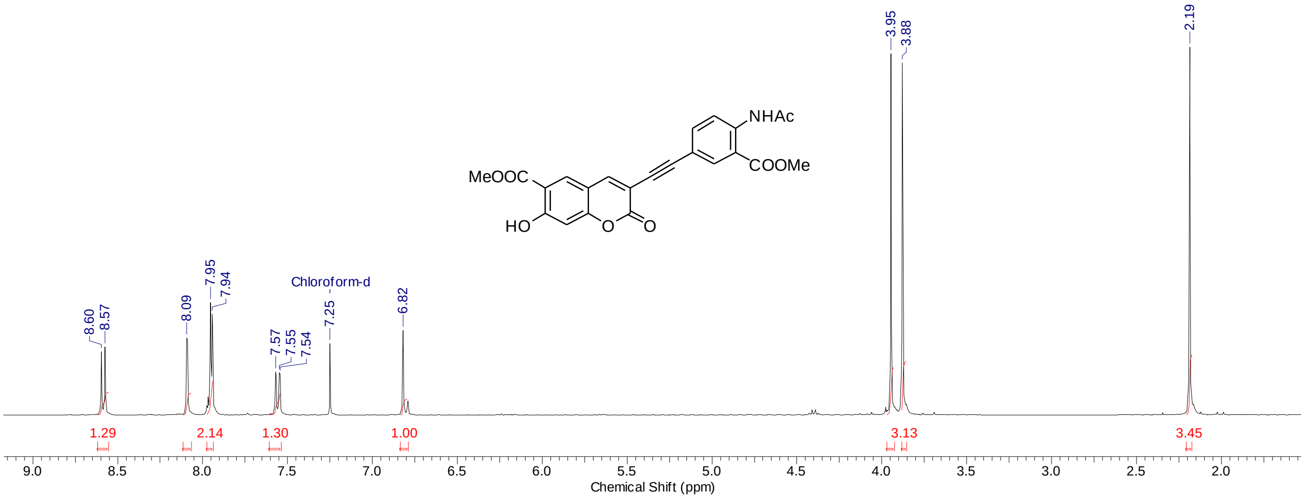

^{1}H-NMR spectra for compound 29 in $CDCl_3$

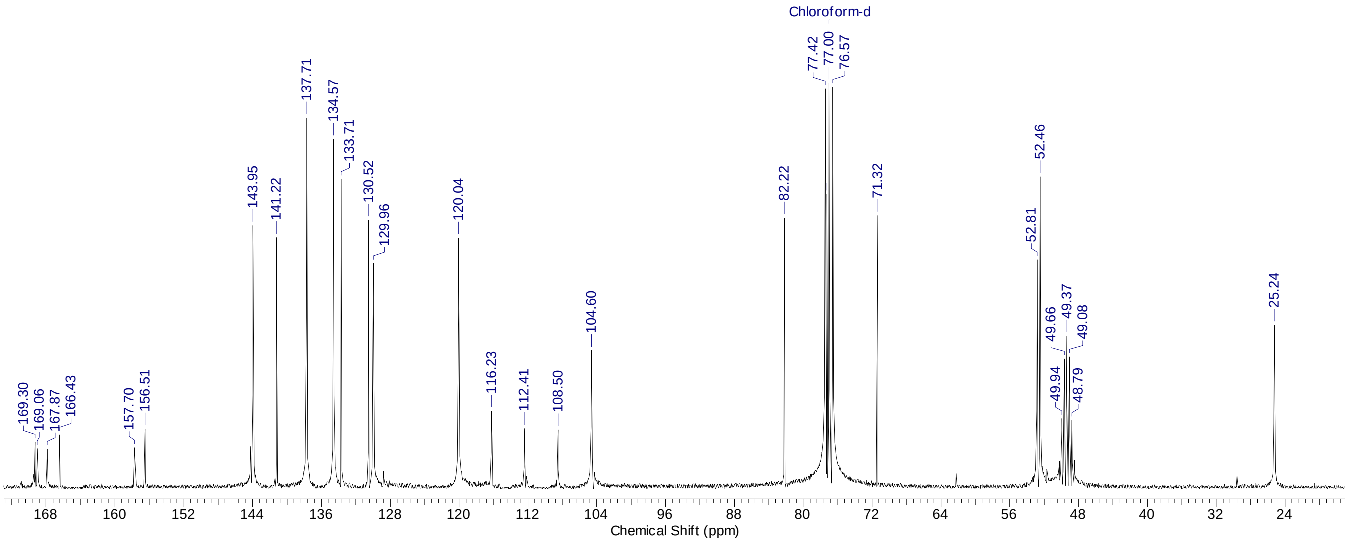

^{1}H-NMR spectra for compound 30 in $CDCl_3$

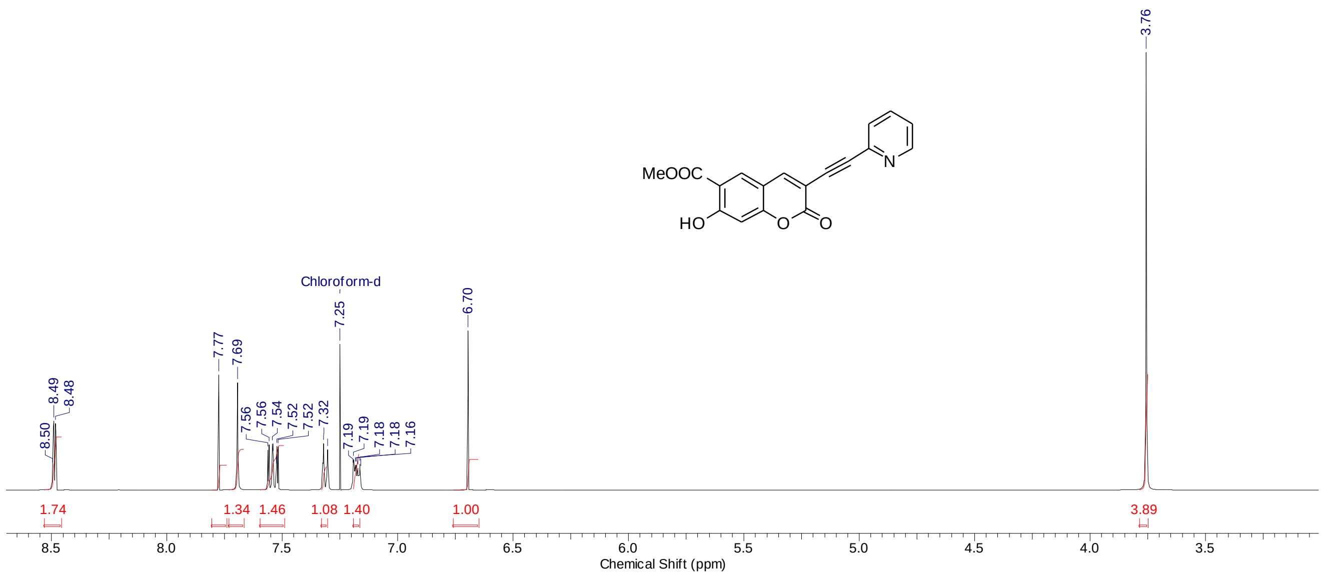

^{1}H-NMR spectra for compound 30 in $CDCl_3$

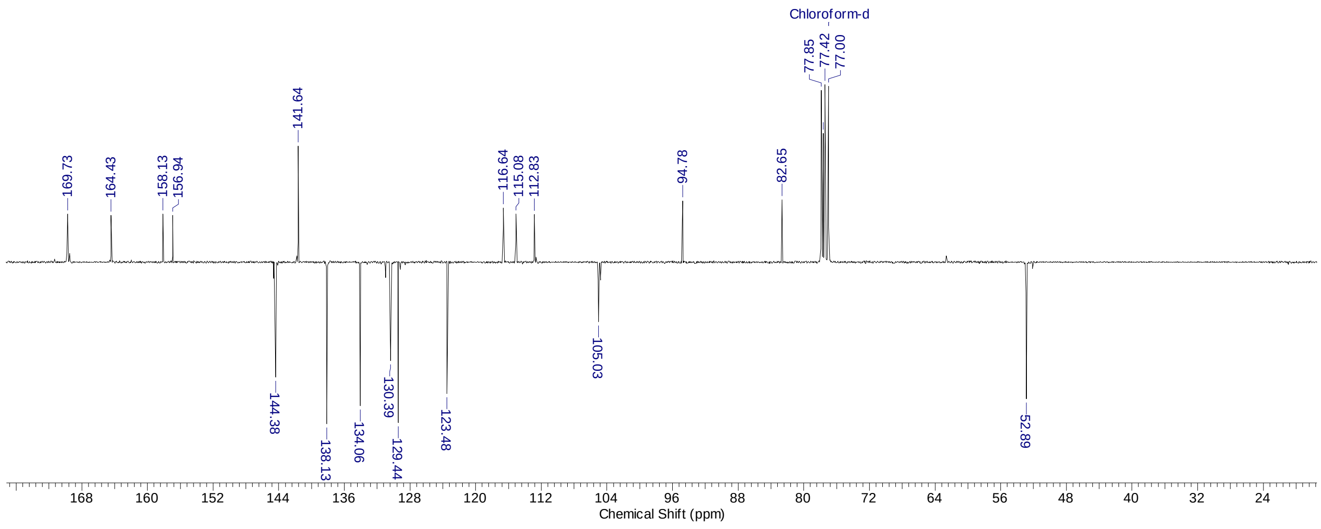

^{1}H-NMR spectra for compound 38a in $CDCl_3$

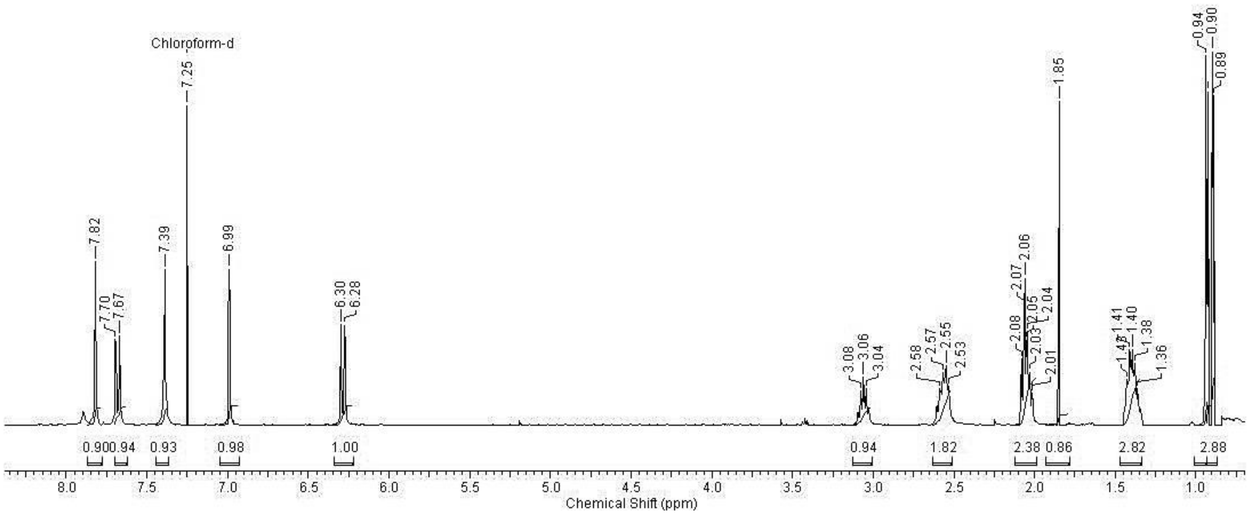

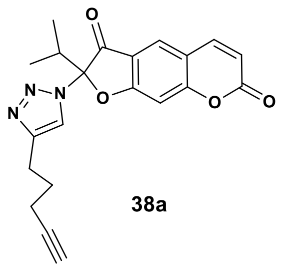

38a

^{13}C-NMR spectra for compound 38a in $CDCl_3$

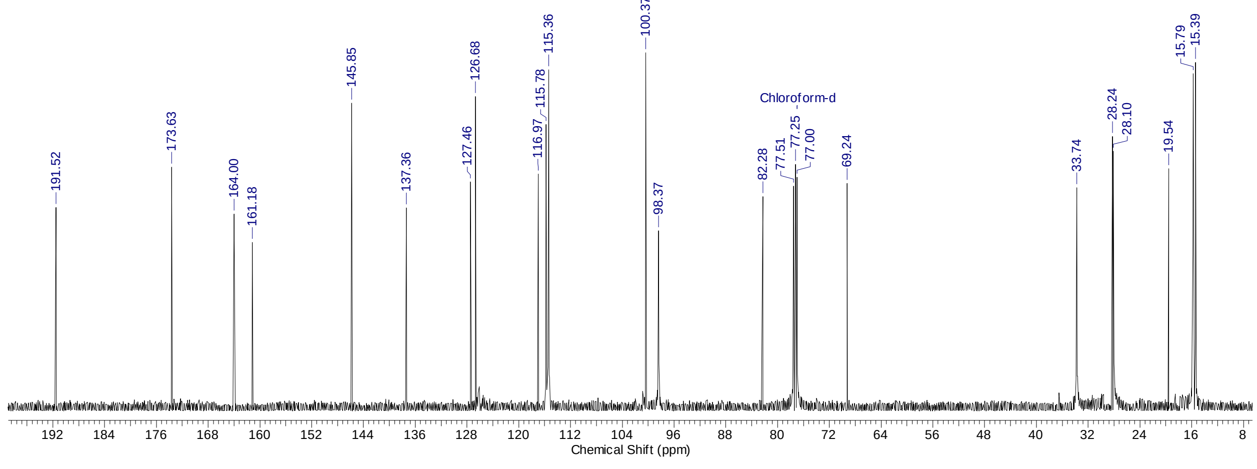

^{1}H-NMR spectra for compound 38b in $CDCl_3$

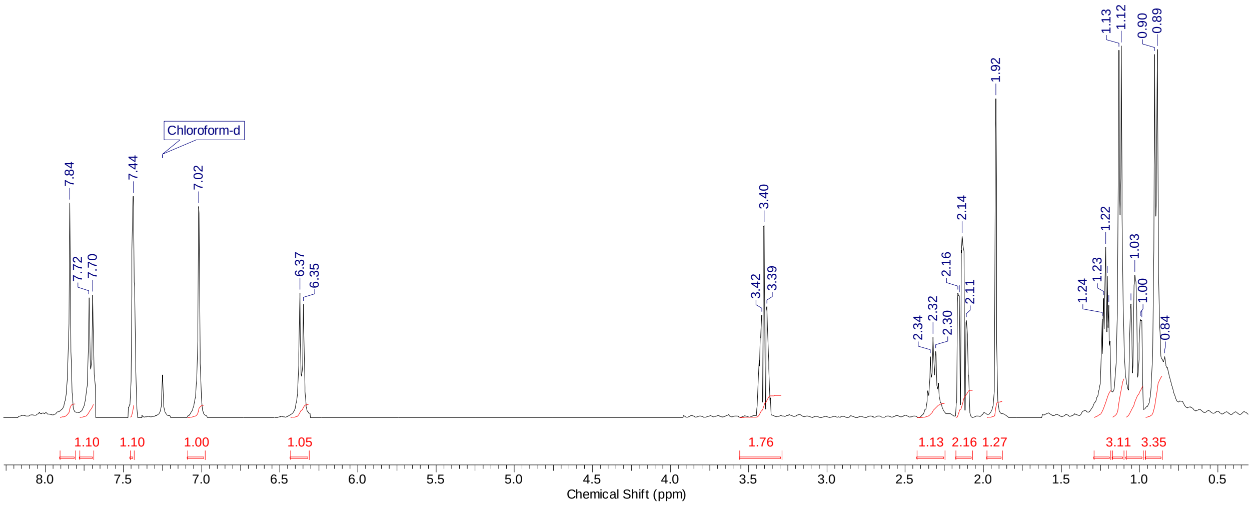

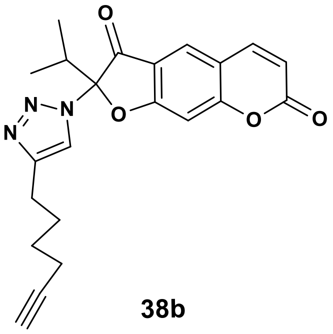

38b

^{13}C-NMR spectra for compound 38b in $CDCl_3$

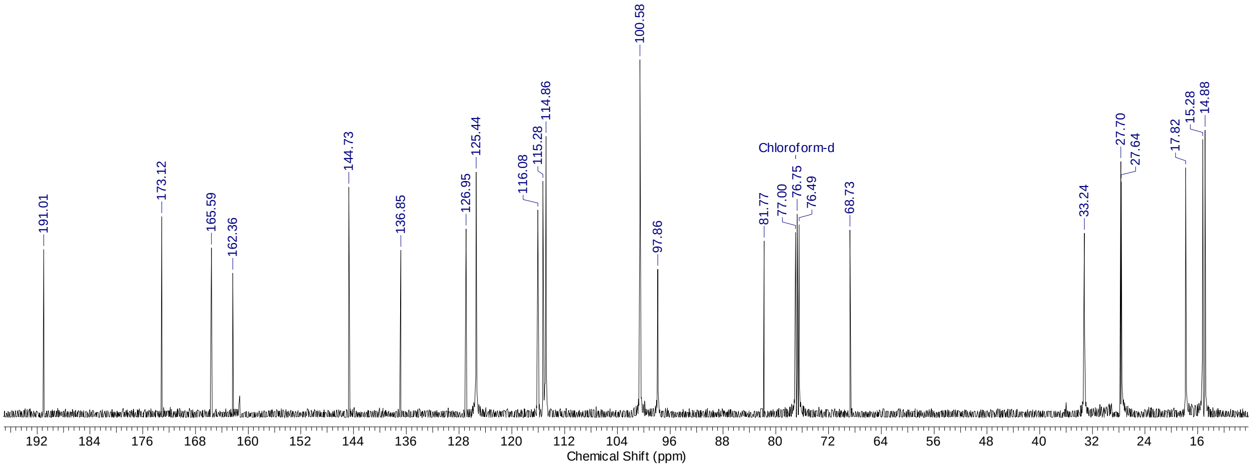

^{1}H-NMR spectra for compound 38c in $CDCl_3$

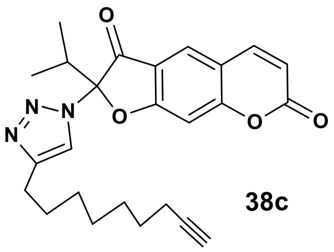

38c

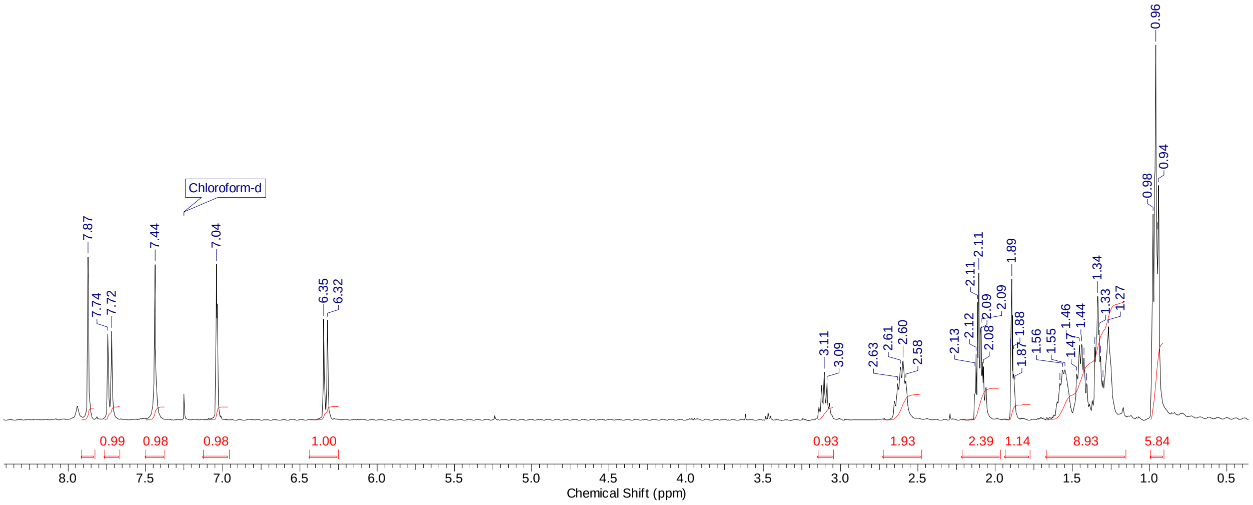

^{13}C-NMR spectra for compound 38c in $CDCl_3$

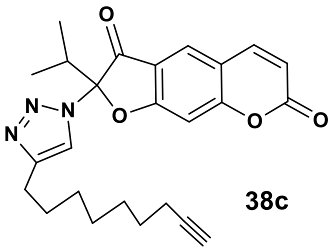

38c

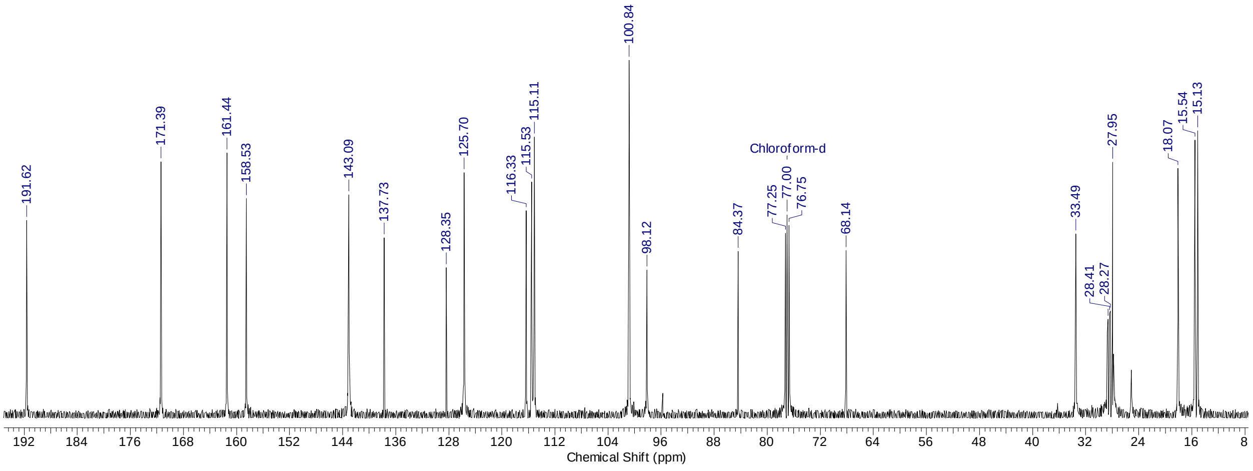

^{1}H-NMR spectra for compound 37a in $CDCl_3$

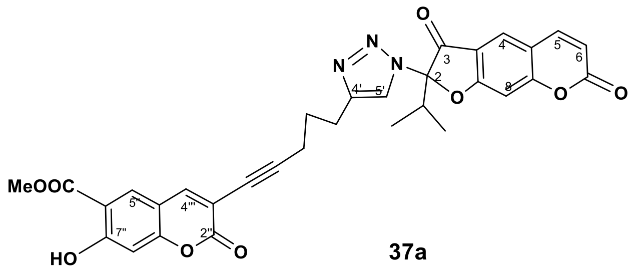

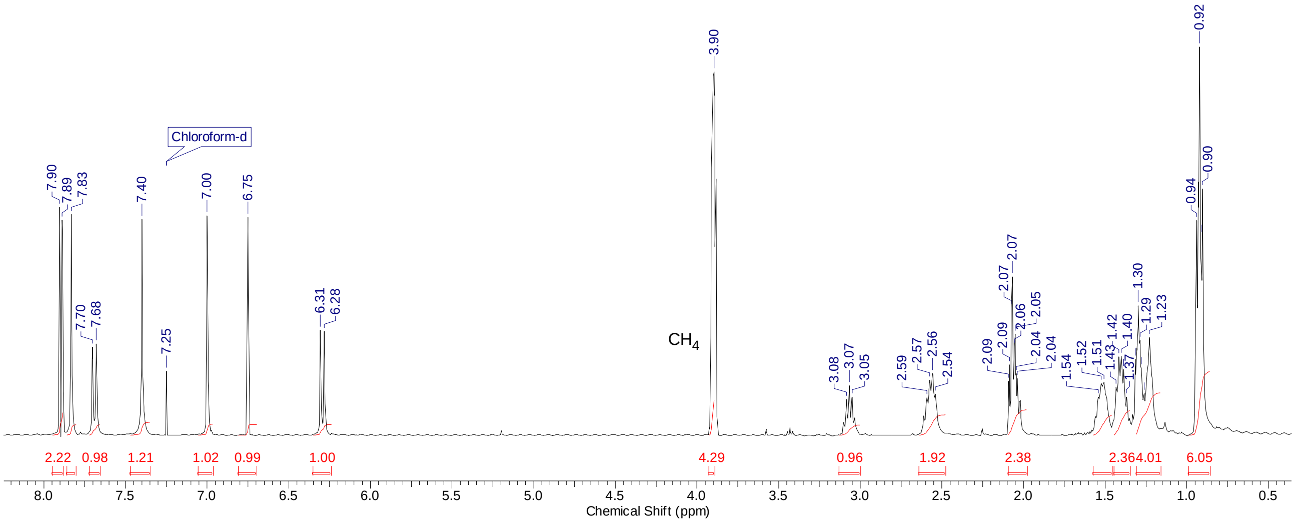

^{13}C-NMR spectra for compound 37a in $CDCl_3$

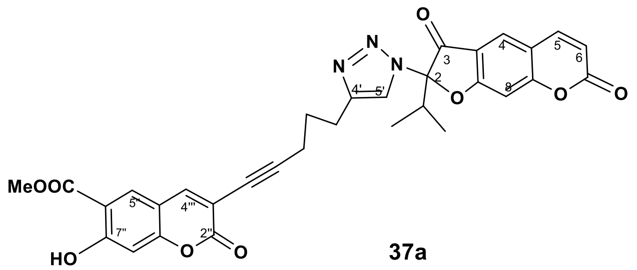

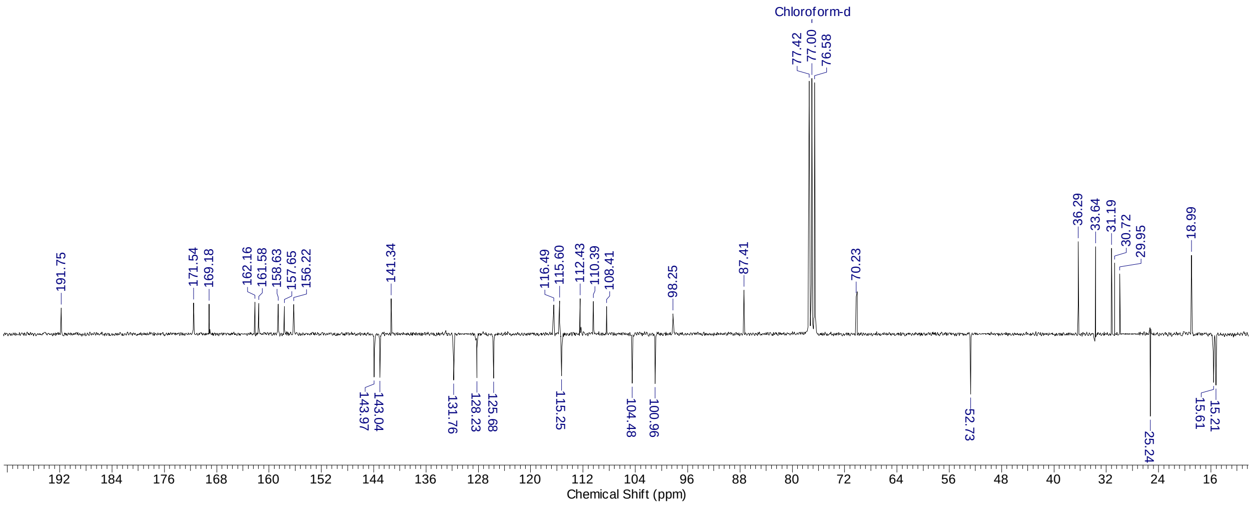

^{1}H-NMR spectra for compound 37b in $CDCl_3$

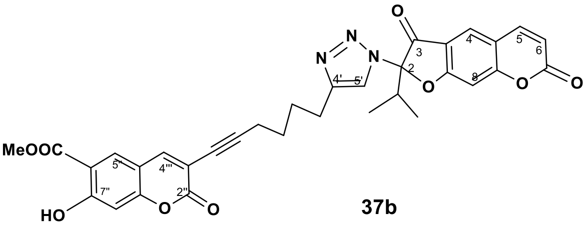

37b

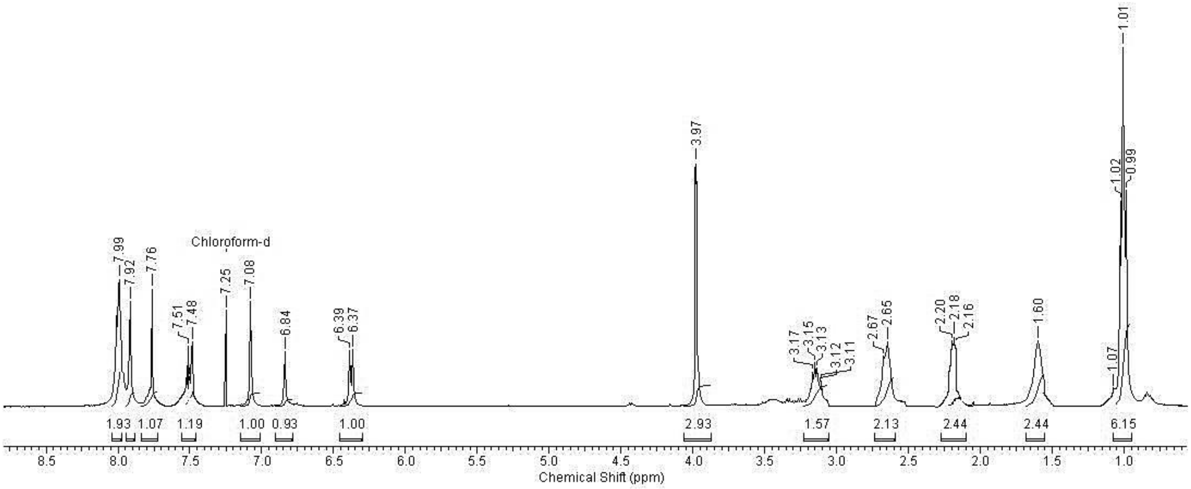

^{13}C-NMR spectra for compound 37b in $CDCl_3$

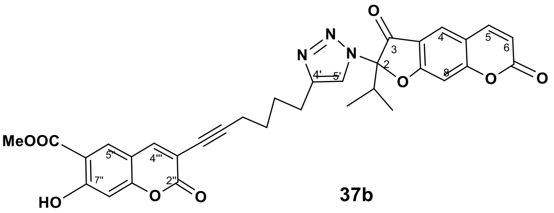

37b

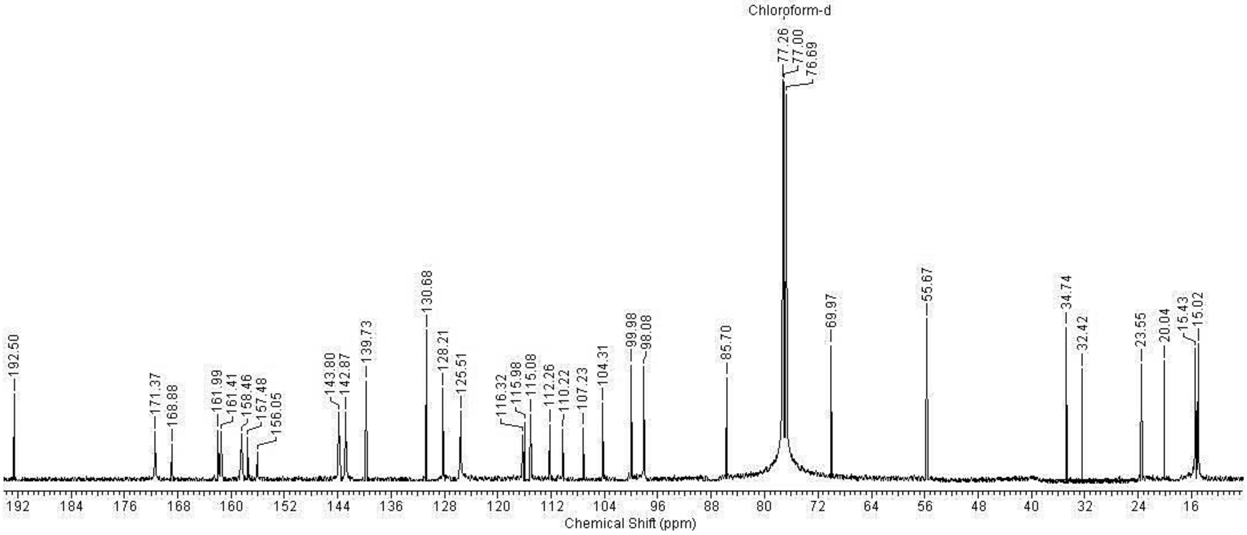

^{1}H-NMR spectra for compound 37c in $CDCl_3$

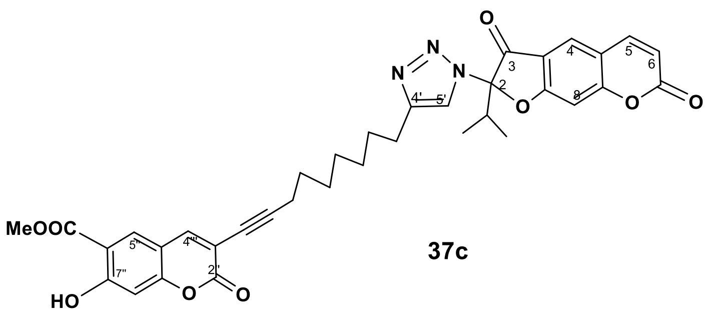

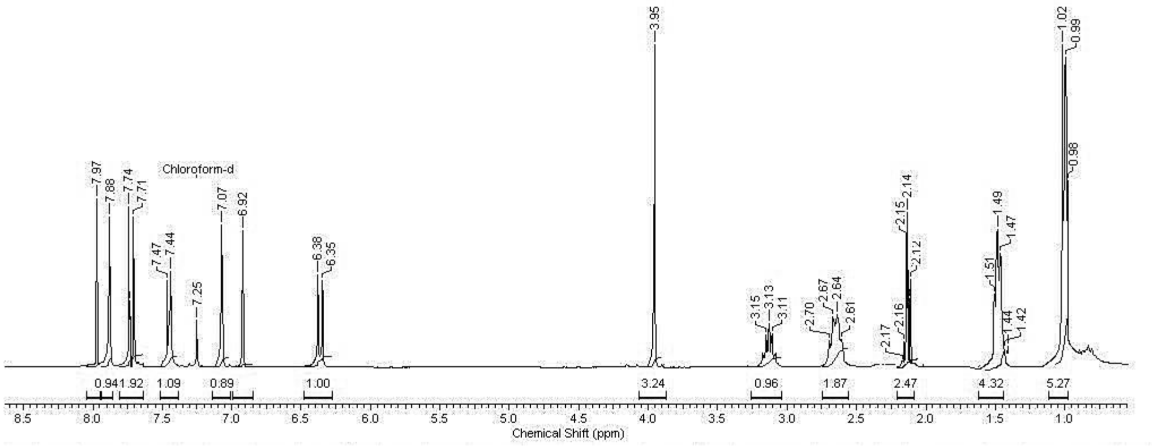

^{1}H-NMR spectra for compound 37c in $CDCl_3$

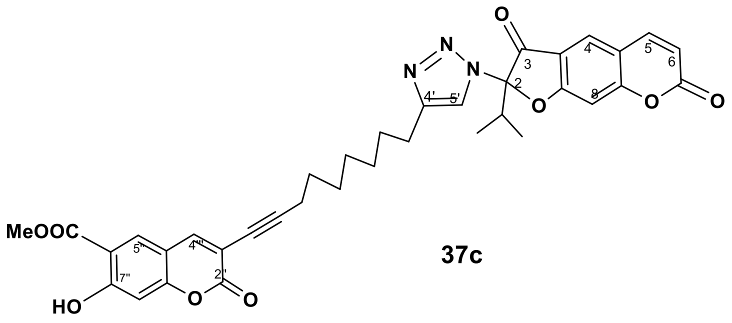

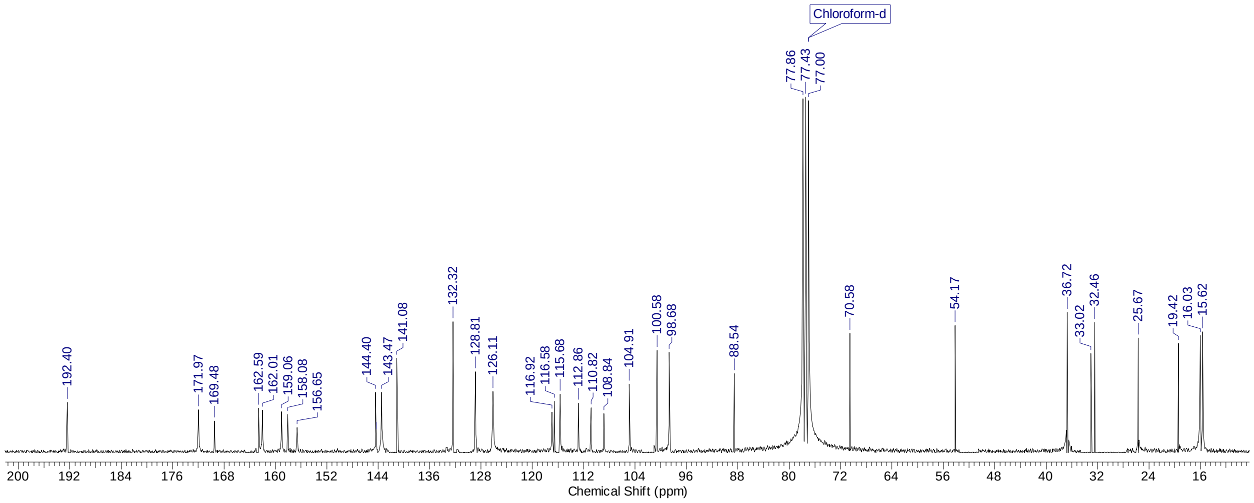

Supplement: Supplementary file 1 [file molecules-24-02126-s001.pdf]
